# Magnesium Infusion on Dental Implants and Its Impact on Osseointegration and Biofilm Development: A Review

**DOI:** 10.1055/s-0045-1806958

**Published:** 2025-04-23

**Authors:** Dyah Anindya Widyasrini, Mutiara Annisa, Siti Sunarintyas, Lakshman Samaranayake, Widowati Siswomihardjo

**Affiliations:** 1 Department of Dental Biomaterial, Faculty of Dentistry, Universitas Gadjah Mada, Yogyakarta, Indonesia; 2 Doctoral Study Program, Faculty of Dentistry, Universitas Gadjah Mada, Yogyakarta, Indonesia; 3 Oral Biosciences, Faculty of Dentistry, University of Hong Kong, Sai Ying Pun, Hong Kong; 4 Dr DY Patil Dental College and Hospital, Dr DY Patil Vidyapeeth, Pimpri, Pune, India

**Keywords:** magnesium, dental implant, coating, osteoinductive, antibacterial

## Abstract

Dental implants have gained global popularity as a treatment option for tooth loss. The success of dental implants depends on their optimal integration into the tissues of the alveolar bone and the periodontium. However, several factors can hinder the proper osseointegration of implants, with the growth of biofilm on the implant surface and subsequent peri-implant infections being significant concerns. To overcome this challenge, researchers have explored the incorporation of antimicrobial agents onto metallic implant surfaces to mitigate biofilm growth. Ideally these agents should promote osteogenesis while exhibiting antibacterial effects. Magnesium (Mg) has emerged as a promising dual-function implant coating due to its osteogenic and antibacterial properties. Despite several studies, the precise mechanisms behind osteoinductive and antimicrobial effect of Mg is unclear, as yet. This review aims to collate and discuss the utility of Mg as a dental implant coating, its impact on the osteogenic process, potential in mitigating microbial growth, and prospects for the future. A comprehensive literature search was conducted across several databases and the findings reveal the promise of Mg as a dual-function dental implant coating material, both as a standalone agent and in combination with other materials. The antibacterial effect of Mg is likely to be due to its (1) toxicity particularly at high concentrations, (2) the production or reactive oxygen species, and (3) pH modulation, while the osteoinductive effect is due to a complex series of cellular and biochemical pathways. Despite its potential both as a standalone and composite coating, challenges such as degradation rate, leaching, and long-term stability must be addressed. Further research is needed to understand the utility of Mg as an implant coating material, particularly in relation to its antibacterial activity, osseointegration, and longevity in the oral milieu.

## Introduction

The demand for missing tooth replacements is on the rise, reflecting growing concerns about oral health. Dental implants have emerged as a popular solution for rehabilitating tooth loss due to their favorable outcome and increasing affordability even in the developing world. Over the past three decades, the use of dental implants has seen a steady increase. 1 2 A relatively recent study conducted in the United States revealed a significant rise in dental implant usage, with rates increasing from 0.7% in 1999–2000 to 5.7% in 2015–2016. Furthermore, it is projected that dental implant use will continue to rise from 5.7 to 23% by 2026. 3

The successful osseointegration of dental implants into the alveolar bone plays a critical role in ensuring their long-term stability and functionality. 4 5 6 Conversely, poor osseointegration can lead to the formation of fibrous tissue or weak junctional bone, resulting in infections and eventual implant failure. 7 8 9

**Fig. 1:**
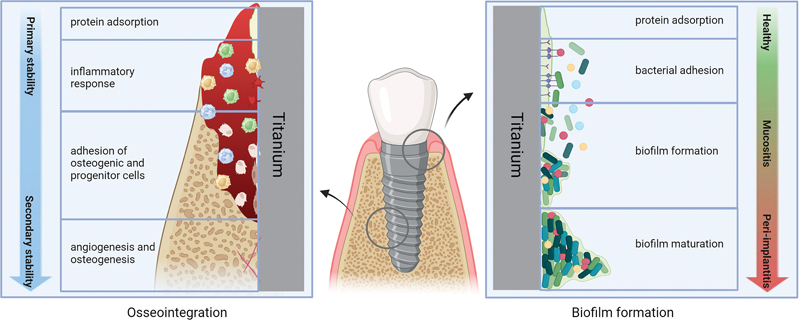
The process of osseointegration and biofilm formation in a dental implant (schematic).

Osseointegration begins immediately after implant placement through mechanical fixation, ensuring primary stability. Blood from the marginal tissue fills the implant bed, forming a pellicle on the implant surface that promotes protein adhesion and cellular activity for new bone deposition to achieve secondary stability. 10 11 Simultaneously, plaque biofilm begins forming upon implant insertion as pioneer bacteria such as *Streptococcus, Actinomyces, Neisseria, Prevotella,* and *Veillonella* species, aided by a salivary protein layer. 12 13 Progressive biofilm maturation can lead to peri-implant mucositis and progress to peri-implantitis with alveolar bone inflammation ( Fig. 1 ). 14

Therefore, minimizing biofilm development is essential to ensure successful bone deposition. 15 16 As both functions are necessary for implant longevity and functionality, developing a dual-function strategy that enhances osseointegration while preventing bacterial biofilm formation is considered important. 6 16 17

Implant surfaces must be tailored to optimize tissue integration and reduce microbial adhesion, as surface properties play a key role in long-term retention. 14 17 18 Consequently, there has been considerable interest in improving the surface properties of dental implants to prevent failure, with surface coatings being the key strategy. 19 Coatings can provide osteogenic and/or antibacterial functions also, enabling controlled release of active agents. 6 14

Many researchers have utilized hydroxyapatite (HA) as an implant coating due to its excellent biocompatibility and osteoconductivity. However, its lack of antibacterial properties remains a limitation. 20 21 Conversely, silver (Ag) is frequently used for its strong antibacterial activity, but its cytotoxicity raises concerns about long-term biocompatibility. 22 Recent studies have shifted toward multifunctional coatings that integrate a single agent capable of simultaneously enhancing osseointegration and reducing bacterial load. 17 18

Magnesium (Mg) holds significant potential as a multifunctional coating material for dental implants due to its dual role in bone remodeling and antimicrobial activity. 23 24 As the fourth most abundant cation in the human body, with half of it stored in mineralized bone tissues, Mg is inherently biocompatible and considered safe for *in vivo* applications. 25 26 It plays a crucial role in HA crystal formation and growth while also regulating bone cell functions, making it essential for osseointegration. 26 Moreover, Mg has been shown to inhibit bacterial growth 27 28 and reduce biofilm formation, addressing one of the key challenges in implant longevity. 29 30 With its unique ability to enhance bone integration while simultaneously providing antibacterial effects, Mg emerges as a promising candidate for advanced dental implant coatings.

In this review, we provide an overview of the use of Mg as a dental implant coating. We aim to elucidate the mechanisms through which Mg acts as an osteoinductive and antibacterial agent while exploring its potential applications. Furthermore, we discuss the prospects and challenges associated with the use of Mg in clinical settings either as a standalone material or incorporated with other chemicals.

## Dental Implant Coating Systems

The surface properties of dental implants play a crucial role in their success by serving as a substrate for microbial adhesion and interacting with body tissues to influence biological responses. 13 14 18 Surface modifications are widely employed to enhance implant surface properties and prevent implant failure, and they have been extensively explored. 19 These modifications primarily aim to improve osseointegration between the implant surface and the surrounding bone, but they have also been expanded to include additional functions such as enhancing antibacterial performance. 6

Some surface modifications involve extensive alterations to improve implant and soft tissue attachments, corrosion, and wear resistance, among other characteristics. 31 32 33 34 These advancements in implant surface modification have been driven by the need for accelerated osseointegration, decreased incidence of peri-implantitis through biofilm reduction, and ensuring long-term implant stability. 33 35 However, integrating multiple features that work synergistically and selecting appropriate surface modification methods with long-term functionality pose significant challenges. 33 36

In recent years, coating materials have been widely employed to modify implant surfaces in both research and the biomedical implant industry. 18 Coating involves the application or spreading of a substance over a substrate, creating an additional layer on the surface. 37 By adding a functional layer through coating, the implant surface characteristics, such as chemical composition, charge, wettability, and roughness, can be significantly altered, thereby affecting cell interactions. 6 Furthermore, surface coatings can enhance interfacial biocompatibility upon contact with body fluids and serve as vehicles for active delivery while improving material corrosion protection. 34 38 This system allows for controlled release of active agents. 14 Such coatings are generally classified as either (1) conversion coatings or (2) deposited coatings. 32

### Conversion Coatings

*In situ* grown coatings produced through particular interactions between specific environments and the base material are typically known as conversion coatings. 32 Because of the chemical or electrochemical process—and sometimes combined with force and heat—that creates a specific environment, an oxide layer is usually formed on the surfaces of the metal substrate. 32 34 39 The geometry of the substrate may change because the oxide layer grows simultaneously inwards and outwards. The resulting layers exhibit inorganic characteristics. 32

With this coating method, studies have reported excellent adhesion strength to the surface because the coating was grown *in situ* . 32 34 39 Conversion coatings serve as an adhesive layer or a coupling agent before the application of deposited coatings. 40 41 As pretreatment, conversion coatings enhance the adhesion of the deposited coating layer. 40 On the contrary, conversion coatings are considered an effective way to improve the corrosion resistance of the metal substrate. 31 39 This type of coatings can be achieved by methods such as plasma electrolytic oxidation/microarc oxidation, 41 42 43 44 ion implantation, 45 46 chemical conversion, 47 48 and hydrothermal treatment. 4 48 49 50 51

### Deposited Coatings

Deposited coatings refer to *ex situ* coatings where the coated surfaces are not involved in the formation of the coating. 52 The materials that compose deposited coatings are flexible, whether they are metal, organic, or inorganic. In this type of coating, intermolecular forces including hydrogen bonds or electrostatic forces and mechanical forces ensure the binding force between the substrate and the coating layer. Because the adhesion of deposited coatings to the substrate is lower than that of conversion coatings, deposited coatings are usually used as the functional layer and placed as the outermost layer of coatings. 32 39 This drawback may lead to the fast release of coatings. 53

Deposited coatings are widely used to achieve more complex biomedical functions. In such situations, different materials are used, such as blending organic and inorganic materials or incorporating metallic particles or ions in organic or inorganic materials. 3 42 54 55 56 57 To maximize the effect or achieve multifunctional coatings, multilayered coating approaches are sometimes used. 7 31 51 58 Many studies have used degradable material as the base of deposited coatings. 54 58 59 60 61 This may suggest that the deposited coating layer will degrade over time as the designated functions are in effect. Currently, the coatings on implant materials are deposited using techniques such as physical vapor deposition, 62 chemical vapor deposition, 62 electrophoretic deposition, 41 42 54 60 pulse laser deposition, 56 plasma spraying, 57 63 dip coating, 55 64 or layer-by-layer (LbL) assembly 31 61 65 methods. 18 66

## Mg as an Osteoinductive Agent

One of the crucial functions of a modified surface is to interact with the extracellular environment and initiate osteoinductive cell responses, including cell proliferation, adherence, and differentiation. The process of osseointegration involves promoting osteogenesis by differentiating osteoinductive progenitor cells into mature osteoblasts while delaying bone resorption and osteoclastic activity. 35 67 68

After implantation, osteoblasts on the damaged pre-existing bone surface are activated and lay down bone on the raw bone surfaces, a process known as indirect osteogenesis. Additionally, some osteoblasts are recruited to the implant surface too, resulting in direct osteogenesis where bone formation occurs from the implant surface. 69

Studies published between 2009 and 2023 that investigated Mg incorporation as dental implant coatings are shown in Table 1 . In four of these studies osteogenic activity assays were conducted *in vivo* , 44 57 70 71 while the remaining studies were *in vitro* investigations. 41 44 45 46 48 56 57 63 64 72 73 74 75 76 77 78 79

**Table 1:** Studies related to Mg coating incorporation as an osteoinductive and antibacterial agents in dental implants

No.	Study (year)	Types of Mg	Cells/animal model	Bacterial cells	Application in biomaterials	Findings as an osteoinductive agent	Findings as an antibacterial agent
1.	Hou et al (2023) 57	Zn–Sr–Mg-doped HAp	1. Human embryonic palatal mesenchymal cells 2. Rat animal model	*1. P. gingivalis* 2. *P. nigrescens*	Titanium dental implant coating	ZnSrMg-Hap promoted strongest osteogenesis and bone growth along implant threads.	ZnSrMg-Hap showed greater antibacterial activity against *P. gingivalis* and *P. nigrescens* compared with Zn-HAp and HAp.
2.	Fan et al (2023) 41	MgO on microarc oxidized treated Ti	Human gingival fibroblasts (hGFs)	*P. gingivalis*	Titanium dental implant coating	High hGF survival rate with minimal cell death; MgO coatings did not disrupt cytoskeleton organization.	MgO coatings reduced bacterial adhesion on Ti surface. The antibacterial rate of 60 s electrophoretically deposited MgO was 53% at 24 hours and 71% at 48 hours.
3.	Tan et al (2023) 46	1. Mg 2. Mg/Ag	hGFs	*1. S. mutans* 2. *P. gingivalis*	Glass-fiber-reinforced polyetherketoneketone (PEKK-GF) dental implant coating	Mg/Ag PIII-treated PEKK-GF enhanced hGF proliferation, adhesion, and adhesion-related gene/protein expression.	Mg/Ag group showing superior antibacterial activity (∼80%) compared with Mg-coated implant group (30–35%).
4.	Liu et al (2021) 48	1. Ti 2. Ti–PA 3. Ti–Mg 4. Ti–PA–Mg PA: phytic acid	Human bone mesenchymal stem cells	*P. gingivalis*	Titanium dental implant coating	Ti–PA–Mg enhanced hBMSC adhesion, proliferation, and osteoinductive differentiation.	Ti–PA showed the highest antibacterial rate, while Ti–Mg and Ti–PA–Mg exhibited similar antibacterial activity.
5.	Yin et al (2021) 72	Mg-Fe layered double hydroxide (LDH) modified titanium	hGFs	–	Titanium dental implant coating	LDH films promote early adhesion, proliferation, and collagen expression of hGFs	
6.	Rezaei et al (2020) 63	Ha-Mg double layer	G-292 osteoblastic cell	–	Stainless steel dental implant coating	The coated samples have better biocompatibility levels than the uncoated sample.	–
7.	Zou et al (2020) 73	1. Nanoporous Mg calcium silicate coating (n-MCS) on PEEK (ncPK) 2. Genistein and curcumin co-loaded in n-MCS on PEEK (dncPK)	Rat bone mesenchymal stem cells (rBMSCs)	*1. E. coli* 2. *S. aureus*	PEEK dental implant coating	dncPK exhibited the highest ALP activity, followed by ncPK, with both improving over time and outperforming PEEK.	Antibacterial activity: no reduction on PEEK, ncPK reduced *E. coli* (54.87%) and *S. aureus* (48.71%), while dncPK showed the highest reduction ( *E. coli* 98.59%, *S. aureus* 99.62%).
8.	Lee et al (2020) 64	Combination of epigallocatechin gallate (EGCG) and Mg ions (Mg ^2+^ ) in the metal–polyphenol network formation	human adipose-derived stem cells (hADSCs)	–	Titanium dental implant coating	Enhanced ALP activity and mRNA expression of osteoinductive markers, mineralization of hADSCs, and increase in calcium content.	–
9.	Wang et al (2020) 74	5Sr5Mg-doped hydroxyapatite (5Sr5Mg-HA)	1. Human embryonic palatal mesenchymal preosteoblasts 2. Beagle dog model	–	Titanium dental implant coating	1. Enhanced biocompatibility, ALP activity, and expression of RUNX2, OPN, and OCN. 2. *In vivo* study showed 5Sr5Mg-HA coating achieved the highest bone-to-implant contact ratio.	
10.	Du et al (2019) 75	Mg	Human bone marrow mesenchymal stem cells	–	Titanium dental implant coating	Mg did not improve the osteoinductive ability but inhibited peri-implant osteolysis.	
11.	Zhao et al (2019) 44	Mg-doped titanium dioxide microporous (MgTiO _2_ )	1. Newborn mouse calvaria-derived MC3T3-E1 subclone 14 preosteoblast 2. Left and right femurs of rabbits	–	Titanium dental implant coating	Promote osteoblast adhesion, proliferation, and differentiation through the ERK/c-Fos signaling pathway	
12.	Ren et al (2018) 76	Amorphous Mg phosphate (AMP)	MC3T3-E1 preosteoblast cells		Polyetheretherketone (PEEK) dental implant coating	Improve the attachment of preosteoblast cells and increase bone remodeling	
13.	Yu et al (2017) 45	1. Mg 2. Zn/Mg	1. Rat bone marrow mesenchymal stem cells (rBMSCs) 2. Human umbilical vein endothelial cells (HUVECs)	*1. P. gingivalis* *2. F. nucleatum* 3. *S. mutans*	Titanium dental implant coating	Zn/Mg-PIII surfaces enhanced initial adhesion and spreading of rBMSCs.	Antibacterial activity: Mg group (10–15%), Zn/Mg group improved inhibition up to 50%.
14.	Mihailescu et al (2016) 56	1. BHA:MgF _2_ 2. BHA:MgO	Hep-2 cells	*1. Micrococcus* sp *.* *2. Enterobacter* sp *.* *3. C. albicans* (All strains were isolated from dental peri-implantitis)	Titanium dental implant coating	MgF _2_ and MgO incorporation enhanced cell adhesion and bonding strength of the coating without cytotoxic effects.	1. Minimum inhibitory concentration for BHA:MgF _2_ and BHA:MgO was 125 µg/mL for all tested strains. 2. BHA:MgO showed the strongest antibiofilm activity, inhibiting *Enterobacter* sp *.* , *Micrococcus* sp *.* , and *C. albicans* at various stages. 3. BHA:MgF _2_ hindered early biofilm adhesion of *Micrococcus* sp *.* and *Enterobacter* sp *.*
15.	Pardun et al (2015) 77	1. Combination of HA, TZ-3YS-E powder, and MgO 2. Combination of HA, TZ-3YS-E powder, and MgF _2_	Human osteoblast cells (HOBs)	–	Zirconia dental implant coating	The Mg-containing coatings exhibited better cell proliferation and differentiation than pure zirconia-calcium phosphate coatings	–
16.	Li et al (2014) 70	Mg-incorporated HA	Implant in the distal femurs	–	Titanium dental implant coating	MgHA-coated implants exhibited higher bone-to-implant contact and bone area ratio than HA-coated implants, along with enhanced trabecular parameters and increased osseointegration.	
17.	Galli et al (2015) 71	Mg loaded mesoporous TiO _2_ thin film	Implant in the tibiae of rabbits	–	Titanium dental implant coating	Successful osseointegration and strong implant–bone interface	
18.	Zhao et al (2013) 78	Electrochemically deposited Mg-substituted hydroxyapatite	MC3T3-E1 preosteoblast	–	Titanium dental implant coating	Increased cell viability of MC3T3-E1, ALP activity, and osteocalcin secretion	
19.	Xie et al (2009) 79	Mg _2_ SiO _4_ powder	Canine bone marrow stem cells (MSCs)	–	Titanium dental implant coating	Good adhesion of MSCs, proliferation, and differentiation behavior on the Mg _2_ SiO _4_ coating surface, and high ALP activity even after 21 days	

The mammalian cells used for *in vitro* assays varied. Examples included pre-osteoblast MC3T3-E1 cells, 44 76 78 human gingival fibroblasts, 41 46 72 rat bone marrow mesenchymal stem cells (rBMSCs), 45 73 and various other cell types. Both *in vitro* and *in vivo* studies yielded promising results regarding osteogenic support of Mg-containing coatings. These effects manifested as enhanced adhesion and proliferation of mammalian cells, upregulated expression of osteogenesis-related messenger RNA (mRNA) and protein markers, and an increased bone-to-implant contact ratio.

Several factors influence the effectiveness of Mg as an osteoinductive agent, including the concentration of Mg and its ratio when incorporated with other materials. 57 For example, Rezaei et al 63 demonstrated that a very high concentration of Mg, when combined with HA, resulted in decreased cell proliferation. Other factors that affect the efficacy of Mg include the surface morphology, porosity content, and Mg content of the samples, as these factors can influence the amount and morphology of the calcium phosphate phase that grows on the surface.

The first possible mechanism of osteogenesis mediated by Mg is enhancement of cell adhesion. 44 72 79 Mg ^2+^ ions, at a certain concentration, increase the affinity of integrins (α5β1, β1, and α3β1) to ligands, including the extracellular matrix (ECM), facilitating cell anchorage to the ECM. 80 81 Integrins, which are integral membrane proteins mediating cell–matrix and cell–cell adhesion, play a crucial role in primary cell adhesion by connecting the intracellular actin and ECM. They also regulate cellular responses and cytoskeleton organization. 81

Numerous studies have demonstrated that Mg ^2+^ is involved in integrin–collagen interactions, promoting osteoblast adhesion through integrins and activating focal adhesion kinase (FAK). FAK, as an integrin signal integrator, can directly activate the ERK signaling pathway and promote osteoinductive gene expression. 80 82 To summarize, interaction of Mg ^2+^ ions with integrins leads to osteoblast cell adhesion, which subsequently influences other signaling pathways that promote cell proliferation and differentiation.

**Fig. 2:**
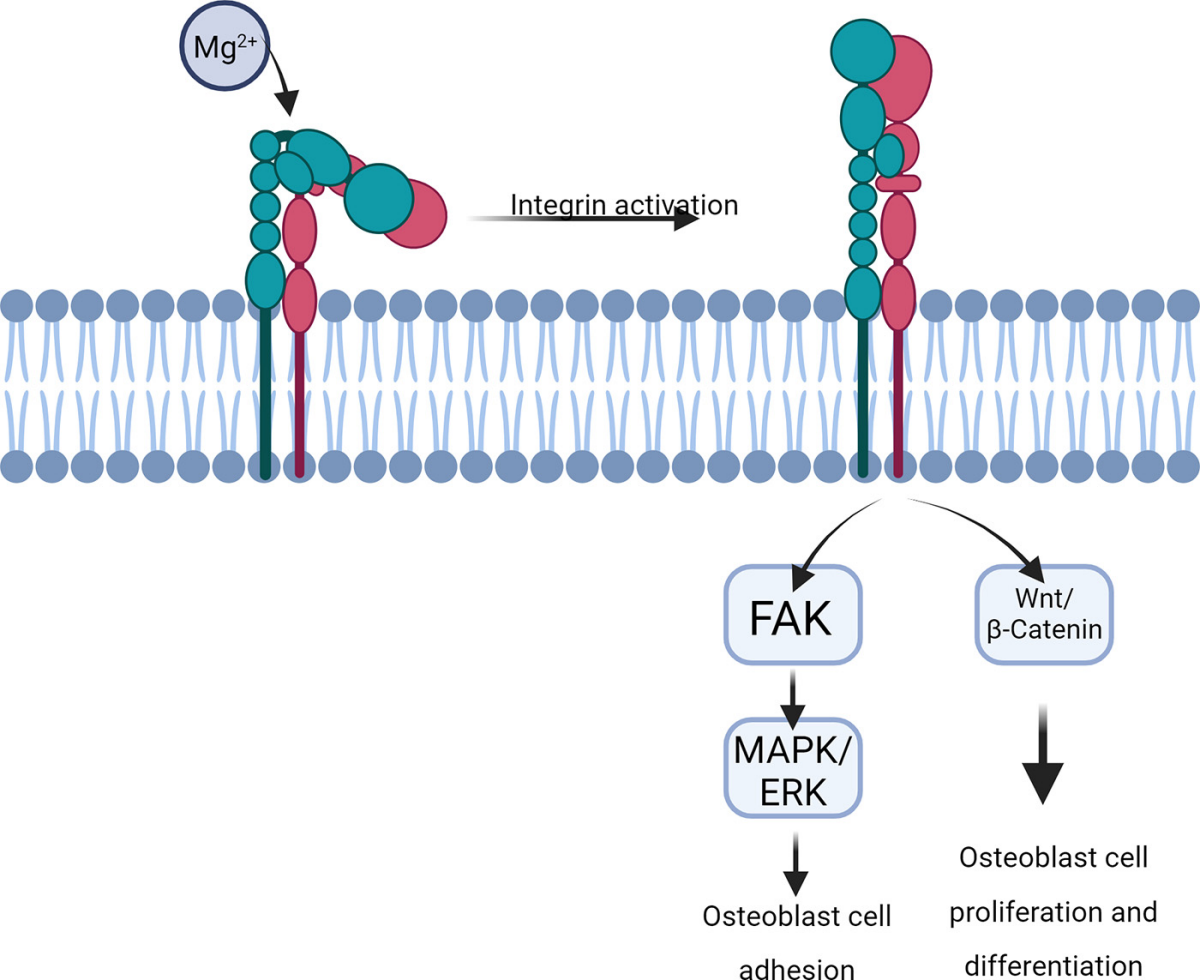
Mg-mediated osteoblast proliferation pathways that occur in alveolar bone during implant placement. 48

The promotion of osteogenesis by Mg ^2+^ ions may also be achieved through the activation of the MAPK/ERK signaling pathway or the stimulation of osteoblast proliferation and differentiation via the Wnt/β-catenin pathway ( Fig. 2 ). The MAPK/ERK pathway is a critical signaling pathway regulating bone development, remodeling, and metabolism. 82 One study demonstrated the involvement of the ERK signaling pathway in the use of Mg-doped titanium dioxide microporous (MgTiO _2_ ) on titanium implants, which promoted osteoblast adhesion, proliferation, and differentiation. 44

**Fig. 3:**
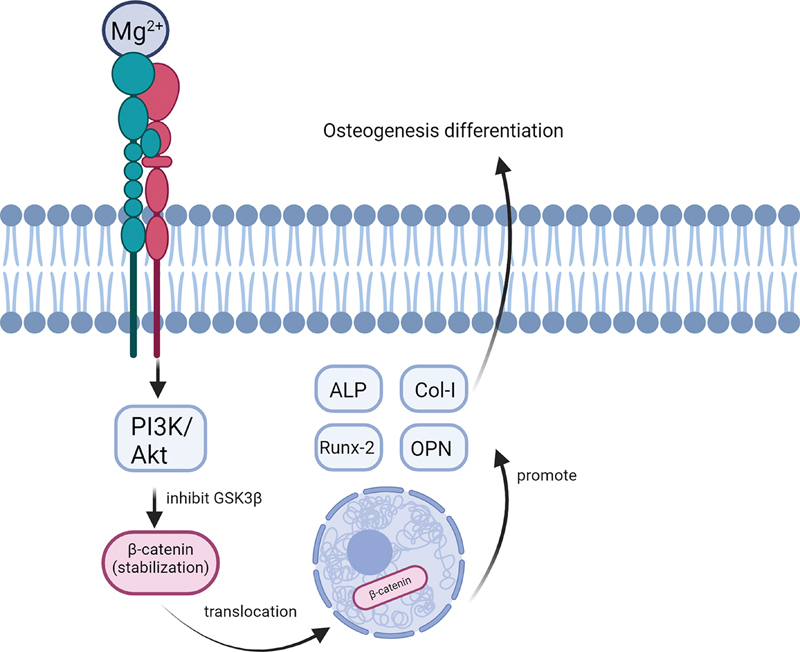
Mechanism of action of Mg in osteogenesis through the PI3K signaling pathway. 48

In addition to the MAPK/ERK signaling pathway, Mg ^2+^ ions stimulate the PI3K/Akt signaling pathways. 64 66 83 This signaling mechanism inhibits glycogen synthase kinase 3 β (GSK3β) which causes β-catenin stabilization and its translocation into the nucleus for gene transcription. This process promotes alkaline phosphatase (ALP) activity along with Col-I, Runx2, and OPN, which leads to osteoinductive differentiation ( Fig. 3 ). 66 83

Some studies have reported an increase in ALP activity with the addition of Mg to a dental implant coating. 64 78 79 83 ALP expressed by osteoblasts is an important enzyme partaking in biomineralization. This enzyme can hydrolyze extracellular inorganic pyrophosphate, generated by the hydrolysis of adenosine triphosphate (ATP), which leads to an increased local concentration of inorganic phosphate (Pi). The latter and calcium ions are thought to accumulate inside matrix vesicles to form amorphous calcium phosphate or HA crystals, which are believed to be the initial stage of ECM mineralization during bone formation. During the osteoinductive differentiation process, the presence and activity of ALP indicate the differentiation of mesenchymal stromal cells toward osteoblasts. 84

Furthermore, Mg upregulates the mRNAs of peroxisome proliferator-activated receptor gamma (PPARG) and glucose transporter 1 in peripheral blood nuclear cells, which plays a critical role in osteoblast growth and differentiation. 57 PPARG plays a role in osteoblasts and osteocytes to regulate bone and fat mass. It has been shown that when such expression is upregulated, osteoblasts and osteocytes produce a high bone mass phenotype and reduce subcutaneous fat mass. 27 This theory could explain the result of the study by Hou et al 57 who found that the addition of Sr and Mg in the coating material improves osseointegration, in which continuous and complete bone coverage was seen on all threads in the cancellous bone with ZnSrMg-HAp compared with Zn-HAP and HAP only. In addition, Sr ^2+^ and Mg ^2+^ ions released stimulate osteogenesis along the implant threads, where the bone growth for osteointegration occurs very early after implant placement.

## Mg as an Antibacterial Agent

The use of Mg as an antibacterial agent in dental implant coatings has not been extensively researched. Out of the 19 publications, only 7 incorporated antibacterial assays to assess the implant coating's ability to combat bacterial growth 41 45 46 48 56 57 73 ( Table 1 ).

The bacterial cultures employed in antibacterial assays primarily consisted of single species associated with periodontal disease, such as *Porphyromonas gingivalis* and *Fusobacterium nucleatum* . 41 45 46 48 57 Notably, only one study utilized bacteria directly isolated from a patient diagnosed with peri-implantitis. 56 Nevertheless, although the antibacterial efficacy of Mg was not consistently positive across all studies, most studies demonstrated this potential.

The antibacterial mechanism of Mg remains poorly understood. Unravelling these mechanisms is essential for harnessing Mg's full potential in biomedical applications, particularly in preventing implant-associated infections. Below, we review several proposed mechanisms, drawing from key studies 24 85 86 87 that suggest pathways through which Mg may exert its antibacterial effects.

### pH Modulation and Disruption of Bacterial Homeostasis

The reduction of bacterial growth in the presence of metallic Mg ^2+^ and aqueous Mg ^2+^ corrosion extracts suggested that the antibacterial activity was associated with the high pH around Mg, generating alkalinity in the surrounding milieu. 88 Under biological conditions, Mg ^2+^ ions that encounter water ions can react with it to form H _2_ , Mg ^2+^ , and OH ^−^ as in the electrochemical corrosion reactions below 89 :

Mg → M ^2+^ + 2e ^-^ (anodic reaction)

2H _2_ O + 2e ^−^ → H _2_ + 2OH ^-^ (cathodic reaction)

Mg ^2+^ + 2OH ^−^ → Mg(OH) _2_ (product formation)

Mg + 2H _2_ O → Mg(OH) _2_ + H _2_ (overall reaction)

Mg(OH) _2_ precipitates when the concentration of localized ions surpasses the saturation limit. This protective layer of corrosive Mg on the surface of biological fluids can inhibit further corrosion. Consequently, the local pH and Mg ^2+^ ion concentration may increase because of the dissolving corrosive layer. Soluble OH ^−^ and Mg ^2+^ ions then quickly diffuse into the surrounding tissues and create a localized alkaline environment. 46 88 90

It is known that most oral bacteria can survive in the pH range of 6 to 8. 86 91 92 Based on these data, it can be postulated that the antibacterial effect of Mg primarily occurs when the pH reaches 9.

The effect of Mg on the suspended/planktonic phase and the attached biofilm phase organisms may vary. The antibacterial effect against planktonic organisms is attributed to the increased alkalinity in the suspended ecosystem while the antiadherent effect for biofilm bacteria is likely to be due to a higher pH value of the Mg-coated surface that mitigates biofilm development. 85

In a cocultured model of *S. epidermidis* and human osteoblasts, Zaatreh et al 93 demonstrated an antibacterial effect and enhanced growth of human osteoblasts on Mg-coated titanium samples. This may be due to several reasons, such as the corrosive dissolution process inhibiting bacterial adherence, osmotic stress on bacterial cells during the initial corrosion phase, the microstructure of the sample surface, an unfavorable rise in pH, or the direct effects of Mg ^2+^ ions. 93

The modulation of pH leads to compromised bacterial homeostasis and inhibits their survival. A plausible explanation is that once bacteria adhere to the Mg-containing surface, a significant amount of H ^+^ produced by the bacteria is used to counter the OH ^−^ produced by the breakdown of Mg. The excessive consumption of H ^+^ disrupts the proton electrochemical gradient within the bacteria's intermembrane space. Since ATP synthesis is driven by the electrochemical gradient of protons, the disruption of ATP synthesis ultimately leads to bacterial death. 85

Furthermore, Nostro et al 94 demonstrated that a higher initial pH has an inhibitory effect on the adhesion of *S. aureus and S. epidermidis* , resulting in impaired biofilm maturation and the formation of poorly structured, thin biofilms. The study also revealed that staphylococci grown at a higher pH had a less hydrophobic cell surface. Such reduced hydrophobicity leads to lower interaction with surfaces, making bacterial adhesion less favorable. 94

### Concentration of Mg ^2+^ Ions and the Bacteriostatic Effect

Higher concentrations of Mg ^2+^ ions appear to inhibit biofilm formation although it could promote bacterial adhesion at low concentrations. 95 Previously, Xie and Yang 96 tested Mg ^2+^ ions as an antibacterial agent against *S. aureus* . They found that Mg ^2+^ has the potential to be membrane-active against *S. aureus* . A minimum Mg ^2+^ dose of 20 mM was needed to significantly reduce *S. aureus* colonies. The membrane permeabilization assays showed that Mg ^2+^ at ≥20 mM caused membrane leakage of *S. aureus* suggesting that Mg ^2+^ may be lethal to *S. aureus* cells by rupturing their membranes. 96 Oknin et al 97 demonstrated that Mg ^2+^ ions at concentrations of 25 mM and above significantly decreased the expression of the two main operons that produce the biofilm matrix, indicating an inhibition of *Bacillus subtilis's* matrix gene expression from forming biofilms. Mg ^2+^ ions may influence the signal transduction for biofilm formation through the Spo0A∼P-dependent pathway, which tightly controls matrix gene expression. 97

Rodríguez-Sánchez et al 98 surmised that Mg ^2+^ ions exhibit profound antibacterial activity against *S. epidermidis* and *Escherichia coli* , and the effect is even more pronounced to sessile/biofilm bacteria. A greater concentration of Mg ^2+^ ions produces a stronger bactericidal effect at constant pH because of the larger osmotic stresses that are generated. The viability of bacteria appears to be affected more by the concentration of dissolved ions than by contact time; however, the longer exposure times exhibited increasing antibacterial effect. 98

Finally, in this context, it is important to note that Mg is an essential element with a very high intracellular concentration. Therefore, the concentration of Mg ^2+^ ions utilized as an antibacterial agent needs to be higher than that found in bacterial cells. 86 The presence of Mg ^2+^ ions reduces the reliance on alkalinity for antibacterial activity. Additionally, moderate concentrations of Mg ^2+^ ions can produce a potent antibacterial effect when combined with alkalinity. 99

### Reactive Oxygen Species Stimulation and Oxidative Stress Initiation

Reactive oxygen species (ROS) production has been proposed as one of the main mechanisms behind the antibacterial activity of nanoparticles, including Mg-based nanoparticles. 41 100 ROSs comprise oxygen-containing chemically reactive particles, predominantly generated within organelles, such as hydroxyl radicals (•OH), reactive superoxide anion radicals (O ^2−^ ), and hydrogen peroxide (H _2_ O _2_ ). 100 101 Physiologically, ROSs are produced through aerobic respiration in bacteria as a response to normal oxygen metabolism and play a crucial role in multiple cellular signaling pathways. 100 102 Although bacteria produce superoxide dismutase to counteract ROS, excessive ROS levels can be detrimental to bacterial cells. 87 103

The surfaces of alkaline earth metallic oxides, including MgO, are known to contain layers of OH ^−^ . Since MgO solutions are naturally alkaline and superoxide ions are chemically stable in alkaline conditions, concentrated O ^2−^ layers may exist on the surface of MgO, along with the hydroperoxyl radical (HO _2_ •). HO _2_ • can generate ROS, leading to the destruction of bacterial cells. 104 Therefore, higher concentrations of MgO nanoparticles may induce ROS production, overwhelming the activity of superoxide dismutase and causing uncontrolled oxidative stress that damages the constituents of the cell membrane. This, in turn, compromises the membrane integrity, resulting in cell necrosis. 87 105

Hayat et al 106 supported a previous theory that MgO nanoparticles increased the rate of bacterial membrane disruption, leading to cellular protein leakage. Gram-negative bacteria exhibited higher leakage of cellular protein contents compared with gram-positive bacteria. Furthermore, a static biofilm method used in the study demonstrated that MgO nanoparticles reduced the potential for biofilm formation in a time-dependent manner. 106 Additionally, the release of Mg ^2+^ ions can also potentially inhibit cellular enzymes, impair mitochondrial respiration, and elevate ROS levels within the mitochondria. 57 100

Several key factors, such as size, shape, surface positive charges, particle dissolution, metal ion release from nanometals and nanometal oxides, and the pH of the medium, can influence ROS production. 107 Nakamura et al 108 observed that smaller sized Mg(OH) _2_ nanoparticles exhibited stronger antibacterial effects. This finding was supported by Huang et al, 109 who co-cultured MgO particles of various sizes with two types of bacteria and found that antibacterial effects increased as particle sizes decreased. They suggested that small particles with larger surface areas generated higher concentrations of O ^2−^ , which could influence ROS production and damage bacterial cell membranes.

## Future Perspectives

Magnesium (Mg) has emerged as a promising material for biomedical applications due to its unique and multifunctional properties. The growing interest in the application of Mg in dentistry, particularly as a coating material for dental implants, is exemplified by the studies in Table 1 . Of the 19 studies identified therein, none utilized pure Mg for dental implant coatings. Instead, Mg was invariably incorporated in combination with other materials, encompassing metals, polymers, ceramics, and biological components. The coating method also varied, employing either conversion coating techniques, deposition methods, or a combination of both.

So far, the combination of Mg with other material types bridges the gap between its opposing roles in osteogenesis and antibacterial function. Numerous studies have combined Mg with metals, polymers, or ceramics, leading to improved osteogenic and antibacterial effects. 45 46 48 56 57 63 64 70 72 73 74 77 Integrating Mg with other metals, such as zinc, strontium, or silver, has shown synergistic effects as antibacterial agents. 45 46 57 However, there are only a few studies that investigate Mg as a standalone implant coating material, making it necessary to further explore its efficacy in terms of osseointegration and antibacterial activity.

While Mg has often been combined with other materials to enhance its performance, the element is effective in its own without further additives as it addresses two critical needs for implants: promoting bone regeneration and mitigating infections. 6 17 However, not all previous studies have assessed both its osseointegration-enhancing and antibacterial properties. Achieving an effective balance between these dual properties remains a key challenge. A central factor influencing both properties is the concentration of Mg used in implant coatings. Too little Mg may not work optimally as an antibacterial agent, while too much may compromise its osteogenic efficacy or lead to unwanted side effects, such as rapid degradation. 45 96 97

Based on one previous study, a Mg surface content of approximately 10% by atomic percentage promotes osseointegration. 110 Further, in another study with a Mg content of 20%, the implant surrogate material showed good adhesion on to human bone marrow stromal cells as well 33 to 37% antibacterial activity against *P. gingivalis* . 48 Another study by Yu et al 45 demonstrated a high proliferation rate of rBMSCs and human umbilical vein endothelial cells, with 10 to 15% inhibition of *P. gingivalis* , *F. nucleatum* , and *Streptococcus mutans* in a sample where Mg was used solely as the coating material. They also noted Mg ion release of 0.018 ppm after 7 days. In other study by Tan et al, 46 after 7 days the Mg ion release of a sample coated with Mg only was 0.25 ppm. It was observed similar proliferation and adhesion rates to the Mg/Ag sample, along with a 30 to 35% antibacterial effect against *S. mutans* and *P. gingivalis* , and a Mg ion release of 0.25 ppm after 7 days. Therefore, determining an optimal Mg concentration is essential for achieving a balance where both functions are maximized without sacrificing one function for the other.

Hence future workers should focus on identifying the optimum Mg concentration to obtain these desired effects through co-culture models using bacteria and mammalian cells. Previous studies have assessed the antibacterial effects and cytocompatibility of other materials in co-culture setups, such as silver and strontium, to define therapeutic windows for dental applications. 111 Similar studies should be conducted to evaluate the concentration-dependent effects of Mg, as no studies have explored this aspect to date.

Additionally, time-dependent or controlled-release coating systems could facilitate the timing of the osseointegration process. For example, antibacterial release should be timed post-implantation and ceased as the focus shifts more toward osteogenesis. 13 112

The selection of coating methods can impact the desired effectiveness and function of Mg coatings and requires further evaluation. The most suitable coating techniques should be carefully chosen in conjunction with the materials involved to tailor the desired effects. 36 For instance, antibacterial agents can be entrapped between layers or incorporated as an essential component of the coating by replacing one of the charged species in LbL deposition, which may lead to multiple layers with differing charges. 53 Moreover, the preparative processes required before dental implant coating should be taken into account. Preceding the deposition of coatings with conversion coating application may enhance osseointegration. 41 42 Therefore, the relationship between the material and the coating process should be thoroughly evaluated when selecting a coating method.

Moreover, despite its well-documented benefits, the precise antibacterial mechanism of Mg is still not fully understood, presenting a notable gap in current research. Unravelling this mechanism is essential to fully exploit Mg's potential as a standalone material for implant coatings. Clarifying these mechanisms could significantly advance the design of more effective Mg-based coatings in clinical practice.

## Conclusion

The review underscores the unique dual functionality of Mg as a coating material for dental implants as it demonstrates both osteointegration capabilities and antibacterial properties. These two roles are synergistic and appear to be vital for dental implant success. Achieving a balance in Mg concentration is a pivotal factor in this context. Additionally, the choice of coating technique is crucial to maximize its dual-role effectiveness. Optimizing these factors could greatly improve dental implant outcomes in the longer term. Therefore, research on the mechanisms underlying each function is crucial.
